# Enhanced human periodontal ligament stem cell viability and osteogenic differentiation on two implant materials: An experimental *in vitro* study

**DOI:** 10.12688/f1000research.129562.2

**Published:** 2023-08-08

**Authors:** Sara F. El Shafei, Shereen N. Raafat, Engy A. Farag

**Affiliations:** 1 Removable Prosthodontics, Faculty of Dentistry, The British University in Egypt, Cairo, Egypt; 2 Department of Pharmacology, Director of Stem Cell and Tissue Culture Hub, Centre of Innovative Dental Sciences (CIDS), Faculty of Dentistry,, The British University in Egypt, Cairo, Egypt; 3 Fixed Prosthodontics, Faculty of Dentistry, The British University in Egypt, Cairo, Egypt

**Keywords:** PEEK, PLDSCs, Sandblasting, Zirconia

## Abstract

**Background: **Surface roughness of dental implants impacts the survival of adult periodontal stem cells and rate of differentiation. This research was conducted to test how human periodontal ligament stem cells behaved on yttria stabilized tetragonal zirconia polycrystals and polyetheretherketone (PEEK) discs with different surface topographies.

**Methods: **Discs roughening was prepared by sandblasting. Stem cells were cultivated on zirconia discs with a polished surface, PEEK discs with a polished surface, sandblasted zirconia discs and sandblasted PEEK discs. Cells viability was assessed after 24, 48, 72 hours. Scanning electron microscopy was used to examine the adherence and attachment of cells. Osteoblastic differentiation capacity was studied by checking the mineralization clusters development through alizarin red S staining and alkaline phosphatase assay. ANOVA and the Tukey post hoc test were used for the statistical analysis.

**Results: **Polished PEEK discs showed lower cell viability, whereas roughened sandblasted zirconia and PEEK discs showed the highest proliferation rates and cell viability percent. The osteogenic differentiation was enhanced for rough surfaces in comparison to polished surfaces. Sandblasted zirconia and PEEK discs showed a markedly increased mineralized nodule development and ALP enzyme activity compared to the polished surface and control.

**Conclusions: **Micro- topographies creation on the PEEK implant surface enhances stem cell attachment, viability, and osteogenic differentiation.

## Introduction

The biological interface between a dental implant and the host tissue in which it is embedded is a critical determinant of clinical outcome. ^1^ To enable osteointegration of the implant, osteoconductive material and coatings are usually used. ^2^ However, in case of significant loss of bone volume (and the unsuitability of bone autografts), the use of osteoconductive materials only will not suffice. The use of osteoinductive material should provide an improved clinical outcome. These materials should stimulate ontogenesis, increase vascularization and hence improve mechanical stability of the implant. ^3^

Human periodontal ligament stem cells (hPDLS) have mesenchymal stem cells characteristics, may regenerate, and develop into a variety of cells, including osteoblasts. Furthermore, their angiogenic potential has been demonstrated *via* the release of vascular endothelial growth factor (VEGF), indicating that they play an important role in implant osseointegration. ^4^

The biocompatibility of titanium with bone tissue makes it a popular material for bone implants. Unfortunately, due to its dark color, titanium implants may become obvious through the gingiva. ^5^^,^^6^ Tetragonal zirconia polycrystals (TZP) on the other hand show outstanding esthetic performance. Additionally, due to their biocompatibility and favorable mechanical properties, TZP has become a pillar material for dental restorations. ^7^ However, from an osteointegration perspective, titanium still shows superiority. ^8^

PEEK is a synthetic polymer which shows potential to be used as an aesthetic dental implant material given its tooth-like color. It possesses exceptional chemical tolerance and biomechanical characteristics making it well-suited for biomedical applications. Notwithstanding, PEEK's bioactivity and osseointegration are also debatable due to shortage of evidence and lack of comprehensive assessment of osseointegration. ^9^

Surface topography modification by lithography, pattern transfer, sandblasting, acid-etching, or plasma immersion ion implantation has been shown to promote cellular adhesion and proliferation. Micro-topographical modifications also create areas in which the bone infiltrates and grows, enhancing implant fixation and stability into bone tissue. ^10^

Sandblasting is one of the most versatile techniques used for implant surface modification. Increased surface area of implants to promote osseointegration is its main benefit. The rougher surfaces also improve osteoblast adhesion and proliferation. ^11^^,^^12^

In comparison to titanium implants, the significance of surface alterations in cell proliferation and the development of osteogenic tissue on zirconia and PEEK implants is much less understood. This study aimed to ascertain the effect of implant material (TZP and PEEK) and surface characteristics (rough versus smooth) on human periodontal ligament stem cell growth and osteogenic differentiation.

## Methods

This study was conducted from 1 ^st^ of February 2022 to 1 ^st^ of November 2022. This study is an experimental *in vitro* study investigating hPDLS behavior using different implant materials with different topographies.

### Disc preparation

Yttria stabilized tetragonal zirconia polycrystals (ZrO _2_ balanced, Y _2_O _3_ < 5.15 mass %, HfO _3_ < 3 mass%, Al _2_O _3_ < 0.5 mass%, SiO _2_ < 0.02 mass %, Fe _2_O _3_ < 0.01, Na _2_O < 0.04) (Bruxzir Shaded, Glidewell) and polyether ether ketone polyetheretherketone (PEEK) blocks were used. The blocks were cut into discs of 1 mm thick and 10 mm in diameter. Four experimental groups of discs were formed according to the type of material and surface treatment: experimental zirconia discs with polished surface (Z); experimental zirconia discs with a sandblasted surface (ZS); experimental PEEK discs with smooth surface (P) and experimental PEEK discs with a sandblasted surface. Using a polishing apparatus (Ecomet 3, Buehler), polished TZP samples were first ground on the two sides with diamond discs (70 m, then 45 m). They were then polished with a polishing cloth, diamond particles measuring 3 and 9 microns, and colloidal silica measuring 0.6 microns. ^13^ PEEK polished samples, however, were produced by first polishing the PEEK disc with 1000 and 2000 grit silicon carbide sandpapers, then finishing with 8000 grit alumina lapping film to offer a smooth surface. ^14^ At 50 mm perpendicular distance on all sides, particles of 110 m alumina (Korox 110, BEGO) were used to sandblast specimens of TZP and PEEK at 5 bar air pressure using a sandblaster (Basic eco, Renfert). All discs were sandblasted for 10 seconds. ^15^ All samples underwent a 10 minutes ultrasonic cleaning procedure using acetone and distilled water before being used in cell culture experiments, followed by a half an hour UV disinfection lamp application on each side of each disc.

### *In-vitro* stem cell isolation and characterization

**Periodontal ligament (PDL) cells isolation and culture**

Under the guidance of the British University in Egypt's Ethical Committee (approval # 22-003) and with patients' (n=3) full agreement and signing an informed consent, three human permanent teeth were gathered from healthy donors at the oral and maxillofacial dental department. The teeth had been extracted for orthodontic purposes. The patients were informed about the study privately and the patient’s data confidentiality was ensured. The patients’ inclusion criteria were; not suffering from any chronic diseases, and average age between 20–40 years. The patients were not further included in the study and were considered as teeth donors only. The selected teeth were free from any carious lesions.

**Figure 1.**
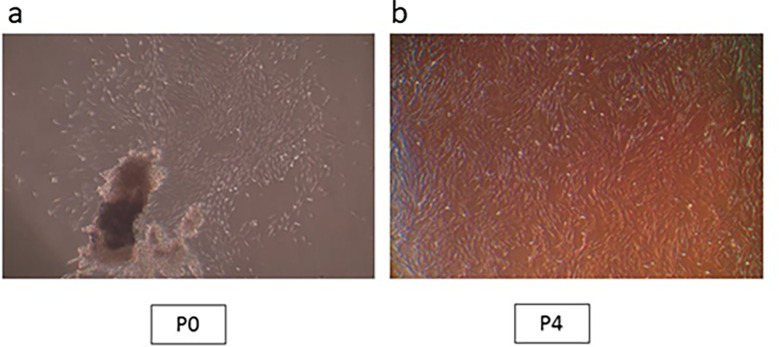
hPLDSCs growth observation under microscope. (a) Cell aggregates at passage 0; (b) Confluent cells at passage 4 (Mag. 40×).

The extracted teeth were kept in Dulbecco’s modified Eagle’s medium (DMEM; Sigma, St. Louis, MO), which received antibiotic doses (300 U/ml penicillin and 300 mg/ml streptomycin; Sigma). Within the first 24 hours from teeth extraction, primary cell culture was performed. Sterile phosphate-buffered saline (PBS, Sigma) was used to disinfect the PDL tissues removed from healthy third molars root surfaces. PBS was then added with antibiotics at escalating doses to provide further protection. Stem cells were isolated from these PDL tissues by using the outgrowth method. ^16^ Smaller pieces of PDL were put onto 35 mm plates (NUNC, Roskilde) with 1 mL of culture media, which was composed of Dulbecco's modified Eagle's medium/nutrient combination F-12 Ham medium (DMEM/F12, Sigma) with 10% fetal bovine serum (FBS, Gibco) added as a supplement and 1% penicillin/streptomycin. 37°C incubation was conducted with 5% CO _2_ in a humid atmosphere. An inverted microscope was used to monitor cell growth and morphology. The cells were trypsinized and sub-cultured once they had reached 80% confluence. Every three days, the medium was changed. All tests were carried out on cells acquired during the fourth passage ( Figure 1a, b).

**Identification of isolated PDL cells as mesenchymal stem cells (MSCs)**

**Cell surface marker identification.** The expression of specific surface markers on the extracted PDLSCs was identified by fluorescence-activated cell sorting (FACS). The cells were collected, moved, and then fixed for 15 minutes in 4% paraformaldehyde. First, 3% bovine serum albumin (BSA; Sigma-Aldrich) was used to incubate the cells before being incubated for one hour with primary antibodies (eBioscience, Thermo Fisher Scientific), produced against CD105, CD90, CD73, CD34, CD45, and HLA-DR for 1 hour. The secondary antibody was applied and left on the cells for 45 minutes at room temperature after being rinsed with wash buffer. After three rounds of washing, a flow cytometer (FACSCalibur, BD Biosciences) was used to evaluate the cells. ^17^

**Multilineage differentiation.** To evaluate the *in vitro* potential differentiation capacity of hPDLSCs, 5×10 ^4^ cells/ml from the fourth passage were cultured in 24-well plate with OsteoDiff media, AdipoDiff media, and CondroDiff media (Human mesenchymal stem cell functional identification kit, R&D Systems) for three weeks to achieve osteogenic, adipogenic, and chondrogenic differentiation, respectively. Alizarin red staining (Sigma-Aldrich) was used to assess mineralization, which demonstrates osteogenesis. Oil red O solution (Sigma-Aldrich) was utilized to find lipid droplet aggregation, which demonstrates adipogenesis. Alcan blue staining (Sigma-Aldrich) was used to identify glycosaminoglycans verifying chondrogenic differentiation.

### Assessment of cell viability, morphology and osteogenic differentiation capacity upon culture on disks

**Cell viability.** Six discs of each group (Z, ZS, P and PS) were put at the bottom of a 6-well plate. Cells were then put to the wells at a density of 30×104 cell for each well. The culturing of cells was on the discs for the following 24, 48, and 72 hours in which cellular viability was evaluated, utilizing methylthiazolyldiphenyl-tetrazolium bromide (MTT) assay (Sigma Aldrich), relative to control cells being cultured in 6-well plates without discs. Absorbance was recorded using a benchtop microplate reader (Thermo Scientific Multiscan). ^18^ The findings were presented as mean viability % relative to controls cultured in the absence of disks.

**Cell morphology.** Cells were seeded in 6-well plates on Z, ZS, P and PS discs as detailed earlier and 72 hours were spent cultivating. Subsequently, 4% glutaraldehyde was used to fix the cells for 2 hours at 4°C. ^19^For critical point drying, samples dehydration was done using increasing ethanol concentrations from 25%,50%, 75%, 95%, to 100%, 5 minutes in each concentration. Samples were subjected to sputter-coating with gold using 15 mA for 4 min (HUMMER 8.0, ANATECH). Discs without cells and those with cultured cells were analyzed by scanning electron microscopy (SEM, Leo Supra 55).

**Osteogenic differentiation assay.** For osteogenic differentiation assay, cells were implanted in a 6-well plate at a density of (30 × 104 cells/well) on disks. The medium was changed the following day to osteogenic differentiation medium, which contains MEM-Alpha, 10% FBS, dexamethasone 100 nm (Sigma Aldrich, Steinheim), ascorbic acid-2-phosphate 200 uM (Sigma Aldrich), and beta glycerophosphate 10 mM (Merck, Darmstadt). ^20^ Differentiation was performed for 14 days. A positive control was achieved using cells cultivated in osteogenic media without discs, whereas a negative control used cells cultured in normal medium (DMEM). Both osteogenic induction medium and basal culture medium were altered twice per week. After 14 days of cultivation, the osteogenic distinction capacity was assessed by the calcium deposition mineralization assay using alizarin red staining and alkaline phosphatase (ALP) activity.

**Alizarin red S assay.** Once calcified, mineralized nodules were observed after 14 days of induction, alizarin red S staining was conducted. In more detail, the medium was aspirated and the cells were fixed with 10% formaldehyde (Sigma, v/v) at room temperature for 15 minutes. After three PBS washes to remove non-attached cells, the plates were stained for 30 minutes at room temperature with 40 mm alizarin red S (Sigma, pH 4.2). To eliminate excess stain, cells were washed 3 more times with PBS before being examined using a light microscope. Additionally, at 405 nm, the absorbance was measured after staining was solubilized in a 10% glacial acetic acid (Sigma-Aldrich) solution. ^21^

**Alkaline phosphatase (ALP) activity assay.** ALP activity of PDLSCs on every specimen was assayed after differentiation for 14 days. Briefly, monolayers were cleaned twice with PBS and then once with 1 mL alkaline phosphatase buffer (ALPB). One mL of ALPB was added to each well and equal volumes of para-Nitrophenylphosphate (p-NPP) (Sigma Aldrich) equilibrated to 4°C were added. Immediately, 50 ul were removed and mixed with same volume of NaOH to stop the reaction. The previous step was repeated every minute for each well till 10 minutes had passed. The rate of accumulation of p-nitrophenolate was plotted for each well and the initial rate of the reaction, a way to express the rate of the reaction, was calculated by obtaining the gradient at the linear phase for each group. Monolayers were then cleaned in PBS and kept at -20°C for total protein quantification. ^22^ Total protein amounts were detected utilizing enhanced BCA protein assay kit (Pierce) based on the manufacturer’s procedures. ^23^ The absorbance at 405 nm was detected by spectrophotometry. The initial rate of the reaction in each group (slope) was divided by protein concentrations (mg).

### Statistical analysis

Each *in-vitro* assay was carried out in triplicate and analyzed across three distinct studies. Graph-Pad Prism v8.1.0 (RRID:SCR 002798) was used to conduct ANOVA test, followed by a pair-wise Tukey's post hoc test, after validating the homogeneity of variance and normal distribution of the data.

## Results

### PLDSCs identification

**Figure 2.**
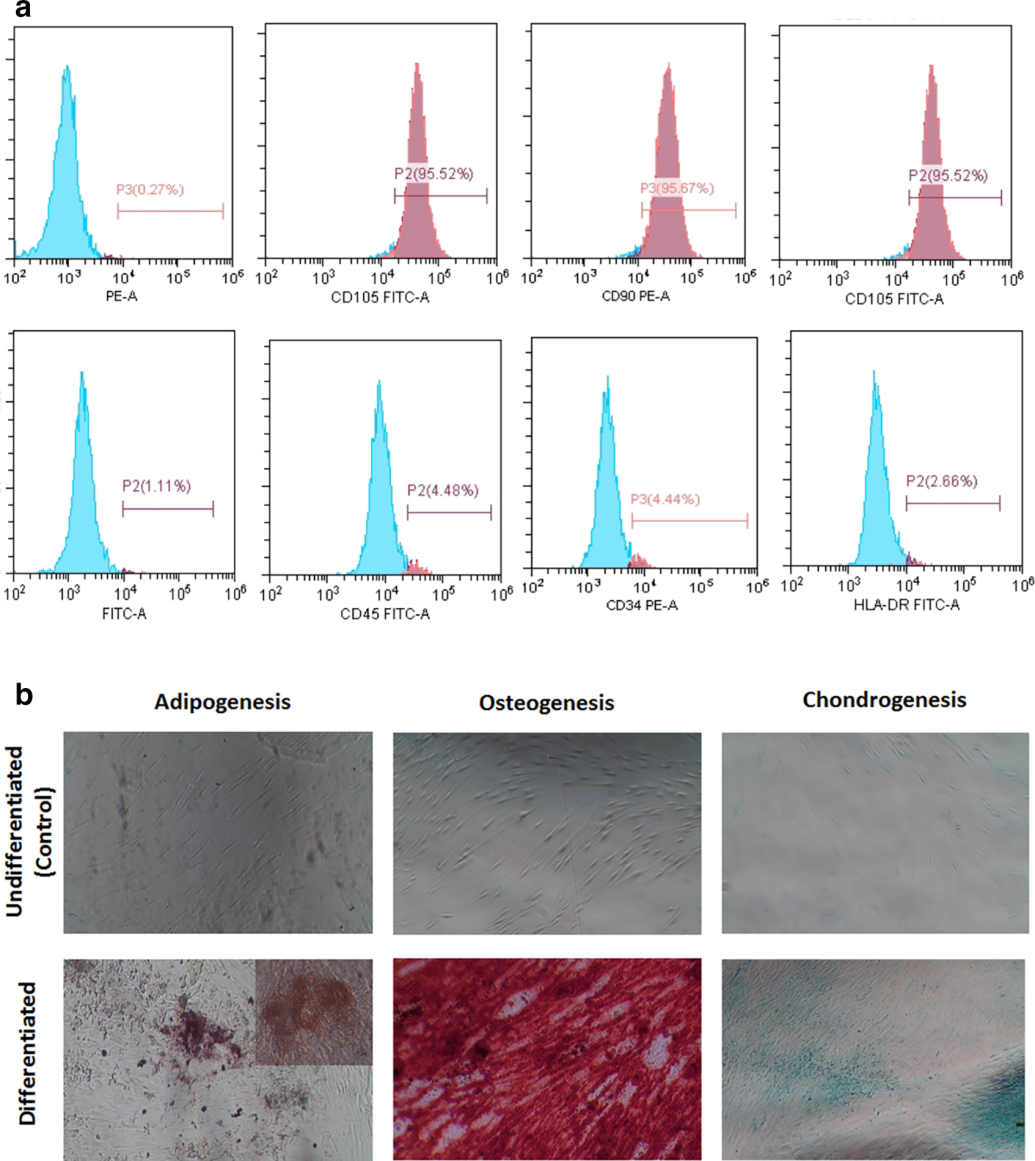
PLDSCs identification (a) Flowcytometry Figures (b) *In vitro* multilineage cell differentiation (Mag. 100×).

According to flow cytometry findings, isolated PLDSCs were CD73 (98.76%), CD90 (95.67%), and CD105 (95.52%) positive; and less than 5% for CD34 (4.44%), CD45 (4.48%), and HLA-DR (2.66%) ( Figure 2a). The rates are provided in respect to the presence of mesenchymal stem cell surface markers. Additionally, hPDLSCs were found to be capable of differentiating into osteoblasts, chondrocytes, and adipocytes ( Figure 2b). PLDSCs differentiated into osteoblasts showed positive red staining of the mineralized matrix using alizarin red S stain, cells differentiated into chondrocytes showed positive staining of the produced glycosaminoglycans via alcian blue stain and cells differentiated into adipocytes showed positive staining of the oil droplets using oil red staining.

### Cytotoxicity assay

**Figure 3.**
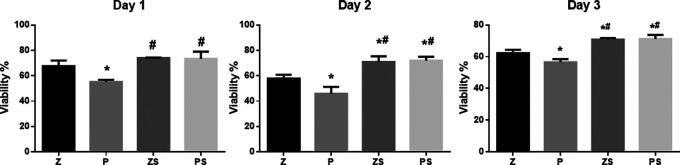
MTT assay. The data are presented as absorbance values (570 nm) at 24, 48, and 72 h of the PLDSCs being exposed to discs. *p significant difference of the respective group in comparison to the Z group, ^**#**^p significant difference of the respective group in comparison to the P group, p values <0.05 are statistically significant. For each material's surface properties, each experimental condition was run in triplicate.

An MTT assay was utilized to determine the viability of the hPLDSCs ( Figure 3). Data are presented after 24,48, and 72 hours of exposure of the PLDSCs to discs and were normalized to the control (cells + medium). Polished PEEK surface discs showed a notably reduced cell viability % in comparison to the three other groups at 24,48 and 72 hours (p<0.05). The sandblasted roughened zirconia and PEEK discs showed increased cell viability % throughout the whole experiment, the roughened PEEK surface possessed significant higher cell viability % compared to the same respective group of smooth polished surface (p<0.001), and the roughened Zirconia discs also showed higher cell viability % compared to the same respective group of smooth polished surface (p<0.05). However, the percentage of cell viability was not significantly different between the roughened zirconia and PEEK discs.

### Cell adhesion and morphology

**Figure 4.**
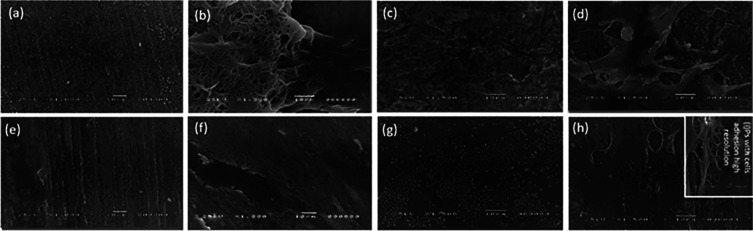
Representative SEM micrographs illustrate (a) polished smooth zirconia surface, (b) cell adhesion on polished smooth zirconia surface, (c) sandblasted roughened zirconia surface, (d) cell adhesion on sandblasted roughened zirconia surface, (e) polished smooth PEEK surface, (f) cell adhesion on polished smooth PEEK surface, (g) sandblasted roughened PEEK surface, (h) cell adhesion on sandblasted roughened PEEK surface, (i) cell adhesion on sandblasted roughened PEEK higher resolution.

The discs’ surface microstructure and stem cell morphology are shown in Figure 4. Cells with extending cytoplasmic processes were frequent and well-observed in smooth polished zirconia discs while no well-defined cells were seen attached to the polished PEEK discs. Cell adhesion and structure of hPLDSCs on roughened sandblasted specimen surfaces revealed the presence of numerous well-attached-type cells with extending stretched cytoplasmic processes.

### Mineralization assay calcium deposition

**Figure 5.**
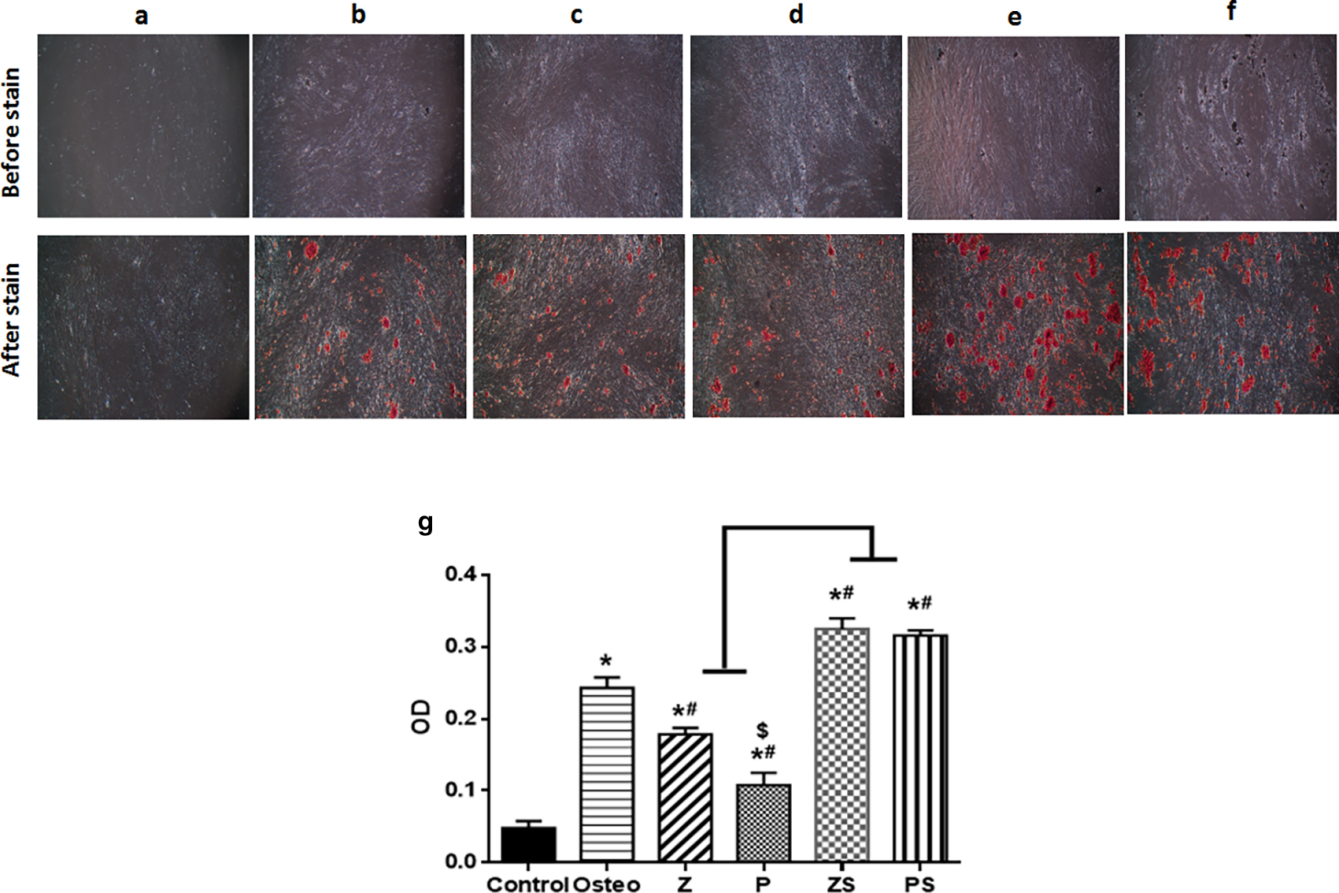
Osteogenic differentiation and alizarin assay: On day 14, as a byproduct of the differentiation of osteoblasts, calcium deposition was examined using alizarin red S staining. (Mag. 40×). (a) Control; (b) Osteogenic medium; (c) Smooth zirconia surface; (d) Smooth PEEK surface; (e) Roughened zirconia surface; (f) Roughened PEEK surface. Every experimental condition was carried out in triplicate for every material surface characteristics. (g) Alizarin red staining data from three independent experiments were statistically analyzed and given as mean standard deviation, *p significant difference of the respective group compared to the control, ^**#**^p significant difference of the respective group in comparison to the osteo group. ^**$**^p significant difference of the respective group in comparison to the Z group. p values <0.05 is statistically significant.

On day 14, extracellular matrix rich in calcium was assessed as a byproduct of the osteogenic differentiation process when stained with alizarin red S ( Figure 5). There were notable variations in between the groups examined; the negative control (cells+normal medium) displayed fewest mineralized nodules amounts; the positive control (osteogenic medium only) exhibited higher amount of calcium deposition (p<0.001) than polished zirconia and PEEK groups ( Figure 5). When compared to the other experimental groups, roughened sandblasted zirconia and PEEK groups possessed greatest grades of mineralized nodules (p<0.001). Average mean values of absorbance in the control, Osteo, Z, P, ZS, and PS groups are 0.048±0.01, 0.24±0.01, 0.1784±0.01, 0.11±0.02, 0.33±0.01, 0.32±0.01, respectively.

### ALP (Alkaline phosphatase) assay results

**Figure 6.**
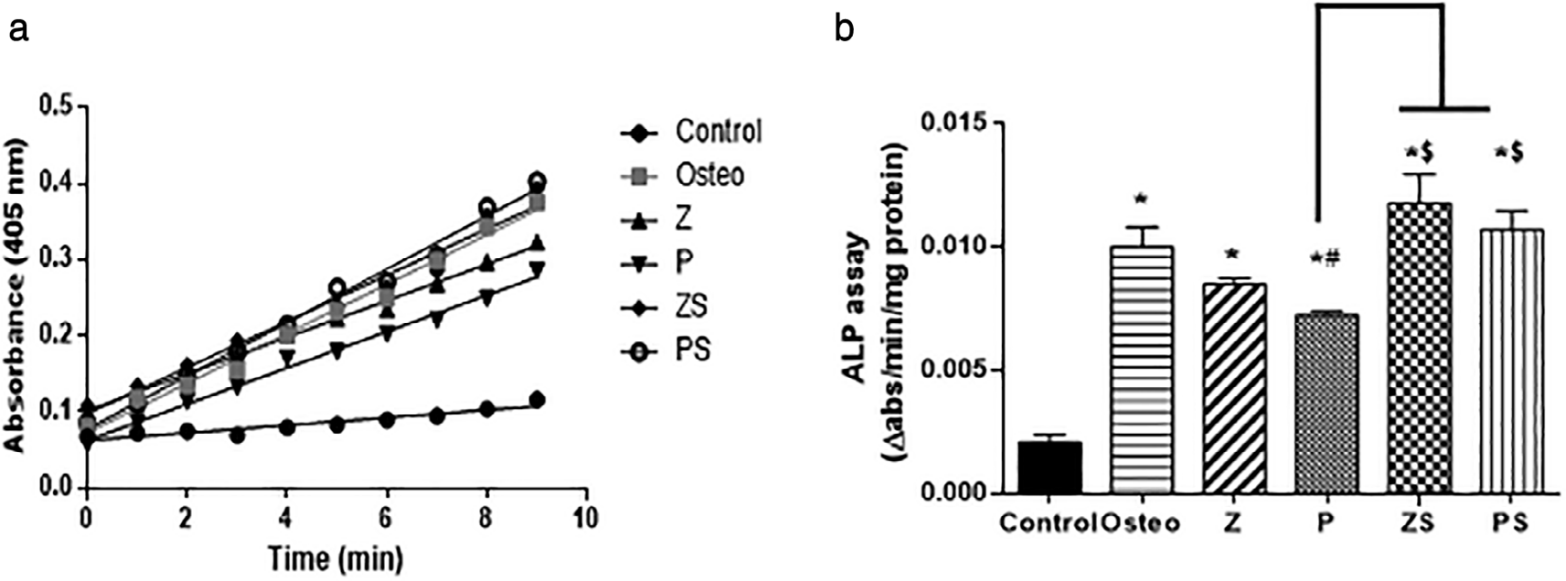
ALP (Alkaline phosphatase) activity after 14 days of osteogenic induction. a) An illustration of an ALP assay's kinetic curve showing how different groups over time accumulated the yellow p-nitrophenolate product. b) The initial rate of the reaction in each group by calculating the slope, values were scaled back to the quantity of total protein. The values revealed as mean±SD of three independent experiments, *p significant difference of the respective group in comparison to the control (cells in normal medium), ^**#**^p significant difference of the respective group in comparison to the osteo group (osteogenic medium), ^**$**^p significant difference of the respective group compared to the Z group. p values <0.05 is statistically significant.

The ALP assay kinetic profile demonstrated the aggregation of the yellow p-nitrophenolate (p-NP) as a growing product among the various groups (shown in Figure 6a). The control group possessed the lowest rate of accumulation, the polished zirconia and PEEK showed lower rate of p-NP accumulation than the roughened sandblasted disks of same respective groups. Statistical analysis was done by calculating the slope of each curve and divided by the total amount of protein in each well. The results showed that the ALP activity at day 14 in cells cultured on the polished zirconia and PEEK discs was significantly lower than the positive control (p<0.05); polished PEEK discs possessed the lowest ALP activity compared to the polished zirconia discs and positive control (p<0.001). Roughened sandblasted Zirconia and PEEK discs indicated most ALP activity in comparison to the polished discs (p<0.001) ( Figure 6b). Regarding ALP activity, no discernible change was found in cells cultured on the roughened sandblasted zirconia discs, PEEK discs and the positive control group, suggesting that roughened sandblasted surfaces can stimulate the osteogenic potential of PDLSCs. Mean values of the rate of ALP reaction (slope) of the control, Osteo, Z, P, ZS and PS groups are 0.0038±0.0002, 0.0125±0.001, 0.0107±0.001, 0.009±0.004, 0.0131±0.001, 0.0135±0.0003, respectively.

## Discussion

Titanium and zirconia are commonly used as dental implant materials, whereas PEEK is widely used in orthopedic research. PEEK, on the other hand, has little biological activity when utilized as an implant, making it unable to successfully osseointegrate with adjacent bone tissue. These flaws significantly restrict the osteogenic properties of PEEK, which is a crucial factor for implants long-term stability. As a result, recent years have seen a surge in research into various methods of modification that might improve PEEK's biological activities, such as cell compatibility, osteogenic activity, and antibacterial capability. ^24^

By enhancing bone-to-implant connection and peri-implant bone density, implant surface modification aimed to accelerate the early phases of osteointegration. ^25^ The surface pattern can be altered by acid etching and airborne particle abrasion, or the surface's physiochemical characteristics can be altered through coating application to enhance the surface's bioactive and osteoconductive qualities. ^26^

Meanwhile, MSCs are capable of self-renewal and cell lineage differentiation into a wide range of cell types, including osteoblasts, chondrocytes, adipocytes, neurocytes, and myocytes. hPDLSCs have been used in this study for the advantages of MSCs as self-renewal, multipotency and immunomodulation, in addition to being a cell population that is conveniently accessible and has the required properties for future clinical use in human studies. ^27^

This study assessed the *in vitro* behavior of hPLDSCs on TZP and PEEK with varying surface topography. The sandblast method was used to micro-roughen the surface; this approach has been utilized for metal implants and is straightforward and affordable. ^28^ To assess the behavior of hPLDSCs on different substrates, we employed different *in vitro* assays including cell viability, morphology and osteogenic potential. Our results showed that polished TZP had better hPLDSCs cell attachment and viability than polished PEEK, whereas sandblasted TZP and PEEK had the highest significant hPLDSCs viability and osteogenic differentiation.

Cell viability was higher on the rough TZP surface than on the smooth surface. Different surface topography presumably modifies the shape and arrangement of the cells' cytoskeletons influencing cell attachment proteins secretion, hence enhanced cell viability and proliferation. ^29^ The viability of hPLDSCs on sandblasted PEEK was significantly higher than on smooth PEEK. Sandblasting produced peaks and valleys on PEEK surfaces resulting in increased PEEK's surface roughness and increased contact angle. ^30^ Danen *et al.* ^31^ proposed that cell adhesion proteins have an arginine-glycine-aspartate peptide sequence that serves as the integrin-binding domain, a protein known to play a role in the adhesion of osteoblasts to biomaterials, and is essential for initial cell attachment, morphology, and proliferation. Because roughened PEEK has a larger surface area, more proteins can be adsorbed on it, resulting in better initial cell attachment and proliferation. Our findings agree with those of Sunarso *et al.,* ^14^ suggesting that roughened PEEK could achieve cell-cell interactions faster than mirror smooth PEEK.

In the current study, the smooth surface specimens of TZP had micro-topography. It displayed advanced cell attachment, a well-adhered cell morphology, and some fibres running between cells, indicating that cells aggregated together. This could be due to the presence of micro-scale granules in the smooth-surfaced TZP. The observed filamentous scaffolding was previously reported to be important in cell adhesion and spread. ^32^

The SEM observations revealed that the cells in the roughened groups exhibited multiple aggregates of cells. Cell attachment samples revealed to be dependent on the finger-like cytoplasmic extensions formation, which was clearly observed on the roughened PEEK specimens in contrast to the polished smooth PEEK. These structures appeared to serve as anchors for the cells to the underlying surface. ^8^ These findings imply that cell rearrangement might result from the sandblasted structure's conversion of the material's physical signals into intracellular biological signals. ^33^

The alizarin red staining and ALP activity assays were used to detect the impact of the tested specimens on the osteogenic potential of hPLDSCs. Our findings revealed that calcium-rich extracellular matrix and ALP activity were significantly higher on sandblasted surfaces than on smooth specimens. All samples showed evidence of calcium nodule development, with differences in aggregation and dispersion based on the topographical type. The mineralization tendency was confirmed by the distribution and density of the nodules. These findings are consistent with Sima *et al*. ^34^ who proved that increased surface granulation, particularly on sandblasted surfaces, indicates that roughened surfaces act as mineralization nucleation centres.

The enhanced osteogenic activity of hPLDSCs could be due to the synergistic effect of micro-topography which may provide a biomimetic surface and influences hPLDSC proliferation and osteogenic differentiation. ^2^ A previous study by Taniguchi *et al*. ^35^ examined how surface roughness affected cultured osteoblast-like cell morphology, proliferation, differentiation and reported that a roughened surface has a greater chance to speed up the calcification process and boost cells' osteogenic ability. Furthermore, the study showed that surface roughness promoted osseointegration of the peri-implant area after application on induced bone defects of rats’ tibiae.

Jung *et al.* illustrated that the kind of cell/material binding seems to be regulated by the implant material. It proved that in osteoblasts, the cell/material binding occur in 60–70% via fibronectin and in 40–50% via laminin. They postulated binding via laminin as a second type of binding and as an enhancement of binding via fibronectin. Fibronectin expression increases over time and with the strongest alteration and surface topography alteration of implant surface. ^36^

Sandblasted zirconia and PEEK surfaces with microscale roughness may provide a suitable niche for hPLDSC proliferation and osteogenic differentiation. A study by Zhu *et al.* ^37^ discovered groove-ridge patterns on the surface of polystyrene that resembled collagen fibrillar architecture more closely in profile and size and that it could be used as a scaffold to provide directed physical cues for osteoblast-like cell orientation, spreading, and directional mineralization.

In contrast with the findings of our study Fukuda *et al.* demonstrated that sandblast treatment alone did not increase osteogenesis in rat-derived MSCs although a slight increase in the osteocalcin level was observed. The study highlighted the importance of combined chemical modifications in addition to surface roughness. ^38^ The non-significant effect of surface roughness in this study may be attributed to the different type of stem cells used and the different sample preparation procedures.

Surface roughness has an impact on cell attachment, proliferation, differentiation, and mineralized matrix synthesis. Our results are in line with those of Lincks *et al.,* ^39^ who found that roughness is an important variable in promoting osteogenic differentiation and appears to affect cell orientation. Furthermore, as surface roughness increases, so does bone development rates and the percentage of surface area in close interaction with bone. In addition, Schneider *et al.* ^40^ studied the influence of implant surface microtopography on the expression of transcription factors regulating osteoblast differentiation. It was observed that there is a relation between cell attachment and surface architecture through gene expression regulation.

To summarize the current study findings, we found that roughened PEEK exhibited high PLDSCs attachment and viability, close to that of roughened zirconia and higher in comparison to the polished PEEK. In addition, higher level of mineralized matrix and ALP activity were observed on the roughened TZP and PEEK. Sandblasted PEEK showed improved osteogenic potential through elevation of the responses of hPLDSCs. The development of micro-topographies on the PEEK surface is a strategy that has the potential to improve PLDSC attachment, viability and osteogenic differentiation. Future efforts should be directed at detailed understanding of the development of more efficient and the study of PEEK modification techniques to enhance accelerated osteogenic differentiation.

## Data Availability

All data underlying the results are available as part of the article and no additional source data are required. Figshare: https://figshare.com/articles/dataset/Raw_data_xlsx/21724385.v1. ^41^ This project contains the following extended data
‐ Supplementary raw data for and statistical analysis of all in vitro assays ‐ Supplementary data of the control figures of flow-cytometry in stem cells characterization ‐ All *in vitro* assays protocols and procedures were done in accordance to; Mesenchymal stem cells assays and applications. https://link.springer.com/protocol/10.1007/978-1-60761-999-4_17 Data are available under the terms of the creative commons attribution 4.0 international license CC BY 4.0.
