# Supplementary material for: Enhanced human periodontal ligament stem cell viability and osteogenic differentiation on two implant materials: An experimental in vitro study
Source: F1000Res. 2023 Aug 8;12:447. Originally published 2023 Apr 26. [Version 2] doi: 10.12688/f1000research.129562.2 (PMC10442589; doi:10.12688/f1000research.129562.2)
Supplement: Peer review history

# Peer review: Reviewer response for version 2

**DOI:** 10.5256/f1000research.154099.r194731

**Published:** 2023-08-21

**Authors:** Sahar El Refai

**Affiliations:** 1 Princess Nourah bint Abdulrahman University, Riyadh, Riyadh Province, Saudi Arabia

I have no further comments than the ones the authors addressed

Is the work clearly and accurately presented and does it cite the current literature?

Yes

If applicable, is the statistical analysis and its interpretation appropriate?

Yes

Are all the source data underlying the results available to ensure full reproducibility?

Yes

Is the study design appropriate and is the work technically sound?

Yes

Are the conclusions drawn adequately supported by the results?

Yes

Are sufficient details of methods and analysis provided to allow replication by others?

Yes

Reviewer Expertise:

NA

I confirm that I have read this submission and believe that I have an appropriate level of expertise to confirm that it is of an acceptable scientific standard.

# Peer review: Reviewer response for version 2

**DOI:** 10.5256/f1000research.154099.r194730

**Published:** 2023-08-10

**Authors:** Samar Darwish

**Affiliations:** 1 Pharmacology and Toxicology Department, Badr University in Cairo, Badr City, Cairo Governorate, Egypt

No further comments to make.

Is the work clearly and accurately presented and does it cite the current literature?

Yes

If applicable, is the statistical analysis and its interpretation appropriate?

Yes

Are all the source data underlying the results available to ensure full reproducibility?

Yes

Is the study design appropriate and is the work technically sound?

Yes

Are the conclusions drawn adequately supported by the results?

Yes

Are sufficient details of methods and analysis provided to allow replication by others?

Yes

Reviewer Expertise:

In vivo studies, inflammation, hepatology, endocrinology, cancer, oxidative stress, kidney, ulcerative colitis

I confirm that I have read this submission and believe that I have an appropriate level of expertise to confirm that it is of an acceptable scientific standard.

# Peer review: Reviewer response for version 1

**DOI:** 10.5256/f1000research.142252.r171165

**Published:** 2023-07-25

**Authors:** Sahar El Refai

**Affiliations:** 1 Princess Nourah bint Abdulrahman University, Riyadh, Riyadh Province, Saudi Arabia

This article is clearly written covering all the details of the material and methods, I acknowledged the standardization in the used technique and the clarity of the results which mostly verified the aim despite figure 2b, (the most right image should have been titled chondrogenesis instead of the 2 images having the osteogenesis title.

 In regards to the discussion, it was sequential and consistent, however would have preferred if the author highlighted the kind of cell/material binding being regulated by cell type and implant material.

 Finally, the addition of opposing opinions to the results addressed in the present research would have enriched the current study outcomes and elucidated all reported literature views.

Is the work clearly and accurately presented and does it cite the current literature?

Yes

If applicable, is the statistical analysis and its interpretation appropriate?

Yes

Are all the source data underlying the results available to ensure full reproducibility?

Yes

Is the study design appropriate and is the work technically sound?

Yes

Are the conclusions drawn adequately supported by the results?

Yes

Are sufficient details of methods and analysis provided to allow replication by others?

Yes

Reviewer Expertise:

Oral cancer

I confirm that I have read this submission and believe that I have an appropriate level of expertise to confirm that it is of an acceptable scientific standard, however I have significant reservations, as outlined above.

## References

[1] : Influence of Implant Material and Surface on Mode and Strength of Cell/Matrix Attachment of Human Adipose Derived Stromal Cell. *Int J Mol Sci* .2020;21(11) : 10.3390/ijms21114110 10.3390/ijms21114110 32526920PMC7312959

# Response

**Published:** 2023-08-02

**Authors:** Sara El Shafei

**Affiliations:** The British University in Egypt, Egypt

Thank you for your efforts in reviewing our manuscript. All the comments have been addressed.

 1-despite figure 2b, (the most right image should have been titled chondrogenesis instead of the 2 images having the osteogenesis title

-Done and corrected.

 2- preferred if the author highlighted the kind of cell/material binding being regulated by cell type and implant material

Added in the manuscript, discussion section and highlighted.

 3- the addition of opposing opinions to the results addressed in the present research would have enriched the current study outcomes and elucidated all reported literature views

Added in the manuscript, discussion section and highlighted.

# Peer review: Reviewer response for version 1

**DOI:** 10.5256/f1000research.142252.r171166

**Published:** 2023-07-14

**Authors:** Samar Darwish

**Affiliations:** 1 Pharmacology and Toxicology Department, Badr University in Cairo, Badr City, Cairo Governorate, Egypt

In this manuscript, the scientists examined and tested how human periodontal ligament stem cells responded to polyetheretherketone (PEEK) discs with various surface topographies and yttria-stabilized tetragonal zirconia polycrystals. The study clarifies how forming micro-topographies on the surface of PEEK implants improves stem cell adhesion, vitality, and osteogenic differentiation. However, there are  few minor revisions that I advise the authors to take them in concern:

**Abstract section**:

1. It is preferred not to write the keywords in their abbreviated forms, such as PEEK and PLDSCs.

**Introduction section**:

1. It is observed that all reference numbers are written after the full stops of the sentences in the whole manuscript. It is preferred to write the reference numbers before not after.
2. 3 ^rd^ paragraph, 2 ^nd^ Line: What is meant by “ *Given their color...*”, please rephrase the sentence to give a clear idea.
3. 4 ^th^ paragraph, 1 ^st^ line: “ PEEK is a synthetic polymer which shows potential...", there is a missing word I think, please revise.
4. 4 ^th^ paragraph, 3 ^rd^ line: the authors mentioned that PEEK's bioactivity and osseointegration are also debatable, please write in more detail what you mean by debatable to clarify your idea.

**Methods section**:

1. PEEK abbreviation refers to which word? Polyetheretherketone or polyether-ether-ketone? Please check.
2. In 'Disc preparation section': please write more details about how yttria stabilized tetragonal zirconia polycrystals were prepared or utilized.
3. In 'Periodontal ligament (PDL) cells isolation and culture': the authors mentioned that " *The extracted teeth were kept in Dulbecco’s modified Eagle’s medium (DMEM; Sigma, St. Louis, MO), which received antibiotic doses (300 U/ml penicillin and 300 mg/ml streptomycin; Sigma).*" Please mention how many doses were given and the dosing frequency.
4. In 'Statistical analysis': " *(RRID:SCR_002798)*" is repeated, mention only once.

**Results section**:

1. Figure 2a: if possible, it is preferred to increase the resolution to make the numbers more clear.
2. Figure 2b: put labels on the 6 different pictures to identify them clearly.
3. Figure 3: again the resolution needs to be increased.
4. Figure 5: there are two rows of pictures, (a, b , c….). What is the difference? Is it the magnification? Please clarify and label them because it is only mentioned that " *(Mag. 40x)*".
5. In the comment on 'Mineralization assay calcium deposition' results, mention the percentage or folds difference calculated between the groups as you can.

**Discussion**: the discussion was well described and covered the study points in detail

1. In the last paragraph: what did the authors mean by this sentence, " *A strategy that has the potential to improve PLDSC attachment is the development of micro-topographies on the PEEK surface, viability and osteogenic differentiation*"? Please rephrase to clarify your idea.

**Reference section**:

1. Some references are preferred to be renewed such as: no 8, 29, 31, 37

Is the work clearly and accurately presented and does it cite the current literature?

Yes

If applicable, is the statistical analysis and its interpretation appropriate?

Yes

Are all the source data underlying the results available to ensure full reproducibility?

Yes

Is the study design appropriate and is the work technically sound?

Yes

Are the conclusions drawn adequately supported by the results?

Yes

Are sufficient details of methods and analysis provided to allow replication by others?

Yes

Reviewer Expertise:

In vivo studies, inflammation, hepatology, endocrinology, cancer, oxidative stress

I confirm that I have read this submission and believe that I have an appropriate level of expertise to confirm that it is of an acceptable scientific standard, however I have significant reservations, as outlined above.

# Response

**Published:** 2023-08-02

**Authors:** Sara El Shafei

**Affiliations:** The British University in Egypt, Egypt

**Thank you for your efforts in reviewing our manuscript. All the comments have been addressed.**

 Reviewer 1:

**Abstract section**:

 It is preferred not to write the keywords in their abbreviated forms, such as PEEK and PLDSCs.

-Corrected PEEK in abstract and keywords  highlighted

-Corrected hPLDSCs in keywords and highlighted

**Introduction section**:

 1-It is observed that all reference numbers are written after the full stops of the sentences in the whole manuscript. It is preferred to write the reference numbers before not after.

-The followed reference numbering style in text is according to the journal submission regulations

 2-3 ^rd^ paragraph, 2 ^nd^ Line: What is meant by “ *Given their color...*”, please rephrase the sentence to give a clear idea.

-Rephrased and highlighted

 3-4 ^th^ paragraph, 1 ^st^ line: “PEEK is a synthetic polymer which shows potential..." , there is a missing word I think, please revise

-Added missing word and highlighted

 4-4 ^th^ paragraph, 3 ^rd^ line: the authors mentioned that PEEK's bioactivity and osseointegration are also debatable, please write in more detail what you mean by debatable to clarify your idea

-Clarified the sentence and highlighted

**Methods section**:

 1-PEEK abbreviation refers to which word? Polyetheretherketone or polyether-ether-ketone? Please check.

-Clarified the word and highlighted

 2-In 'Disc preparation section': please write more details about how yttria stabilized tetragonal zirconia polycrystals were prepared or utilized

-Rephrased to clarify the idea and highlighted

 3- In 'Periodontal ligament (PDL) cells isolation and culture': the authors mentioned that " *The extracted teeth were kept in Dulbecco’s modified Eagle’s medium (DMEM; Sigma, St. Louis, MO), which received antibiotic doses (300 U/ml penicillin and 300 mg/ml streptomycin; Sigma).*" Please mention how many doses were given and the dosing frequency.

Antibiotic-antimycotic drug is added once in the culture medium bottle (500 ml) which is used in the whole study; changing medium for the cells is maximum 2-5 ml / well or flask twice per week.

 4- In 'Statistical analysis': " *(RRID:SCR_002798)*" is repeated, mention only once.

-Removed and mentioned only once.

**Results section**:

1. 1-Figure 2a: if possible, it is preferred to increase the resolution to make the numbers more clear.

2-Figure 2b: put labels on the 6 different pictures to identify them clearly

-Figure 2a. Edited and added in the manuscript

-Figure 2b. Edited and added in the manuscript

 3-Figure 3: again the resolution needs to be increased

-Edited and added in the manuscript

 Figure 5: there are two rows of pictures, (a, b , c….). What is the difference? Is it the magnification? Please clarify and label them because it is only mentioned that " *(Mag. 40x)*".

 - Done and labeled

 In the comment on 'Mineralization assay calcium deposition' results, mention the percentage or folds difference calculated between the groups as you can

 - Mean values of absorbance of the study group are added in the manuscript and highlighted.

**Discussion**:

 1-In the last paragraph: what did the authors mean by this sentence, " *A strategy that has the potential to improve PLDSC attachment is the development of micro-topographies on the PEEK surface, viability and osteogenic differentiation*"? Please rephrase to clarify your idea.

 - Rephrased and highlighted
